# Comparison of experiences of nursing staff and patients before and after move to 100% single-bed room hospital in Australia: mixed methods

**DOI:** 10.1186/s12913-023-09073-8

**Published:** 2023-01-25

**Authors:** Lynette Cusack, Rebecca Munt, Naomi Verdonk, Tim Schultz, Jill Maben

**Affiliations:** 1 Adelaide Nursing School, Faculty of Health and Medical Sciences, University of Adelaide, Adelaide, South Australia Australia; 2 Flinders University, Adelaide, South Australia Australia; 3 University of Surrey, Guildford, UK

**Keywords:** Case study, Mixed methods, Patient experiences, Nurses experiences, Staff expectations, Single-bed room hospitals

## Abstract

**Background:**

There is sufficient and consistent international evidence of issues reported by nurses working in single-bed room environments, requiring a design that is not only comfortable for patients but meets nurses working needs. This paper presents a comparison of nursing staff and patients experience prior to a move to 100% single-bed room hospital in 2016 (Stage 1) and actual experiences after the move in 2021 (Stage 2) in South Australia.

**Method:**

Mixed method case study design. Survey sample of forty-two nursing staff; twelve patient interviews of their experiences of current environment and; thirteen nursing staff interviews of their experiences delivering nursing care in 100% single bed-room environment.

**Results:**

Nurses and patients highlighted single-bed rooms contributed to patients’ privacy, confidentiality, dignity and comfort. As anticipated in Stage 1, nurses in Stage 2 reported lack of patient and staff visibility. This impacted workload, workflow and concern for patient safety.

**Conclusion:**

Patient and nursing staff experiences are interdependent, and implications of single-bed room accommodation are complicated. Future impacts on the health system will continue to affect hospital design, which must consider nurses working needs and patient safety and comfort.

**Supplementary Information:**

The online version contains supplementary material available at 10.1186/s12913-023-09073-8.

## Background

There has been an international trend for hospitals to be built with an increased number of single-bed rooms [1–3]. In keeping with trends towards hospitals with a greater proportion of single-bed rooms, a hospital in Adelaide South Australia, was built with 100% single-bed rooms, vastly different to the structural environment in the previous hospital that consisted of a mix of single and multi-bed room wards. Moving to the new single room hospital potentially required a change of care delivery and working practices for nursing staff. This paper presents a comparison of nursing staff and patients experience prior to a move to 100% single-bed room hospital in 2016 (Stage 1) and actual experiences after the move in 2021 (Stage 2).

A scoping review by Søndergaard et al. [4] identified changes to work practices (less time spent with patients and lonelier practice), increased walking distances, concerns for patient safety, quality of care, and staffing levels in an all single-bed room hospital. Patients, largely indicated a preference for single-bed rooms [3] in particular the en-suite facilities, privacy and control over the environment such as reducing noise and light levels, enhancing their sleep [4]. Other known advantages for patients in single-bed rooms include potential to experience greater confidentiality, better quality of communication between health professionals and patients and families, and enhanced family involvement in patient care [5, 6]. However, both Maben et al. [6] and Søndergaard et al. [4] reported that some patients felt forgotten by nurses, with potential concerns for their safety with others reporting feeling lonely. Similarly, patient safety outcomes, have remained a constant concern reported by nurses in single-bed room environments, in which patient monitoring and surveillance can be more difficult contributing to a potential increased falls risk [1, 2].

We conducted a study in 2016 (Stage 1), in the old hospital, which identified advantages and disadvantages for patients and nursing staff of a pending move to 100% single-bed room hospital, as well as comparison with findings from similar studies in England [1, 2, 6]. Four constructs were derived from the study: physical environment; patient safety and comfort; nursing staff safety; and importance of interaction. Nursing staff highlighted potential perceived disadvantages, which included increased walking and travelling time between patients, missed early signs of deteriorating patients, and risks to nursing staff safety. Patients identified perceived disadvantages as reduced regular contact with staff and other patients and potentially a sense of isolation, particularly for older people [1]. Potential advantages identified by both nurses and patients included reducing patient bed movement around the ward, reduced hospital acquired infections, improved sleep and rest, ease of assisting patients to the bathroom and maintaining patient confidentiality, dignity and privacy [1]. The current study (Stage 2) repeated the same methods as Stage 1 to examine the actual experience of nurses and patients in single-bed rooms in the new hospital.

## Methods

The study followed a mixed method case study design [7] similar to Maben et al.’s [8] study protocol [2]. In Stage 2 (2019-2021) nursing staff and patients from four wards, similar to the wards in Stage 1 (2016), (surgical and medical specialties) were invited to participate. There were two methods of data collection, a survey of nurses followed by semi structured interviews with nurses and patients. However, a delay in data collection occurred between the nursing staff survey (2019) and nursing staff interviews (2021) due to COVID-19 pandemic, which prevented researcher access to wards and nursing staff. Stage 2 Patient interviews occurred pre COVID (2019). Given the time between Stage 1 and Stage 2 survey periods, it is unlikely that a large proportion of nursing participants were included in both stages.

### Nursing staff survey

Nursing staff working on the four wards (*n* = 291) were invited to participate in the electronic survey. Flyers were placed in handover rooms introducing nursing staff to the study. Text messages inviting them to participate were sent, followed by two text reminders. The survey tool (69 items) used by Maben et al. [2] was adapted, with permission, and applied to both Stage 1 [1] and 2. Ten sub-scales of current ward layout, environment and facilities were measured in the survey (Care delivery; Family/Visitors; Infection control; Patient amenity; Patient safety; Physical Environment; Privacy/Confidentiality; Staff amenity; Staff Safety; Teamwork/training), and were derived from a mean of 7 items per sub-scale (range 3 to 14) using a 5-point Likert scale response (S1). Results from Stage 1 were published in 2019 [1].

### Nursing staff and patient interviews

In both study stages, nursing staff working on the four wards were invited to participate in a semi structured interview either face-to-face or telephone. Nursing staff were informed about the interview by a member of the research team attending afternoon handover and through flyers. Nursing staff emailed the nurse researcher if interested in participating. Following signed consent, semi-structured interviews were digitally recorded and conducted by members of the research team who had no clinical or management responsibilities within the hospital. The topic guide (S2), was the same for both stages to enable a comparison. It included recent experience in clinical service at the hospital; staff experience relating to physical environment; impact of ward layout, environment and facilities on direct care, communication and teamwork, documentation and medication tasks, staff and patient safety; patient experiences relating to physical environment; and suggestions for improvements. All thirteen interviews were face to face and occurred in a quiet space on wards. Interviews lasted between 30 and 60 minutes.

Patients receiving care on the four wards were invited to participate in a semi structured interview by research team nursing academics. Clinical nursing staff nominated patients to be approached by researchers to ensure patients had capacity to give informed consent and well enough to participate in an interview. Sixteen patient interviews were sought however, data saturation was reached at 12 interviews. Following signed consent, semi structured interviews were conducted using a topic guide (S3), which was exactly the same for both study stages to enable comparison. Questions focused on patient experiences of current structured environment and ways this related to their overall experience of care, including; experience of being admitted to the ward; feeling comfortable; feeling safe; interaction with nursing staff; and interaction with visitors. Interviews occurred face to face in patients’ single-bed rooms and lasted approximately 30 minutes.

### Data analysis

The characteristics of study population in survey data were analysed using descriptive statistics. Results for the ten sub-scales for Stage 2 were compared against previously collected Stage 1 data using a Student’s t-test in SPSS (v25.0, Armonk, NY: IBM Corp).

Transcripts of nursing staff interviews (*n* = 13) and patient interviews (*n* = 12) were analysed separately by two researchers using reflexive thematic analysis [9]. This included the six phase process of data engagement, open coding and theme development [10]. A degree of deductive analysis was employed to ensure open coding contributed to producing themes that were meaningful to the research questions and enable comparison of data from Stage 1. The two researchers collaborated in the active knowledge of applying the same theoretical assumptions and coding structure from the previous study Stage 1 in the knowledge production. The researchers kept an open mind to the interpretation of any new meanings. Nursing staff and patients data from Stage 1 and Stage 2, were then compared, using similar themes, on the experience prior to a move to 100% single-bed room hospital in 2016 (Stage 1) and actual experiences after the move in 2021 (Stage 2).

### Ethics

Approval by ethics committees: anonymised Human Research Ethics Committee, Protocol No: R20160620 HREC/16/Central Adelaide Local Health Network 227) and The University of Adelaide Human Research Ethics Committee application ID: 33711.

## Results

### Nursing staff survey

A total of 67 participants commenced the survey in Stage 2 although only 42 completed the final section of the surveys (Demographics) a response rate of 14% eligible nurses). All 67 surveys were included in the analysis up until the point that the participant stopped the survey. The demographics from the 42 completed surveys are summarised in Table 1. Twenty-five participants (60%) were registered nurses, while the remainder (40%) were enrolled nurses (*n* = 17).

**Table 1:** Demographics of 42 completed survey responses from Stage 2

Variable	Level	Registered nurses		Enrolled nurses	
		*n*	*%*	*n*	*%*
Gender^a^	Female	22	91.7	15	88.2
	Male	2	8.3	2	11.8
Age^b^	21-30	7	30.4	4	23.5
	31-40	5	21.7	6	35.3
	41-50	5	21.7	2	11.8
	51-65	6	26.1	5	29.4
Hours a week^a^	Up to 29	9	37.5	7	41.2
	30 or more	15	62.5	10	58.8
Pay band^a^	1	12	48.0	5	31.3
	2	10	40.0	5	31.3
	3	2	8.0	0	0.0
	4	0	0.0	0	0.0
	5	1	4.0	6	37.5
Education	In Australia	23	92.0	17	100.0
	Outside Australia	2	8.0	0	0.0
Highest qualification	Diploma (inc Advanced Dipl)	3	12.0	17	100.0
	Bachelor	15	60.0	0	0.0
	Postgrad	7	28.0	0	0.0
		*mean*	*SD*	*mean*	*SD*
Years of experience	Occupation	15.3	9.5	7.5	3.6
	Specialty	9.3	7.4	6.3	4.5
	In this hospital	12.1	8.8	6.8	4.2
	On the ward	7.8	6.2	6.1	4.2

^a^ sample size is 41, ^b^ sample size is 40

### Stage 1 and stage 2 survey comparison of ward layout, environment and facilities

**Fig. 1:**
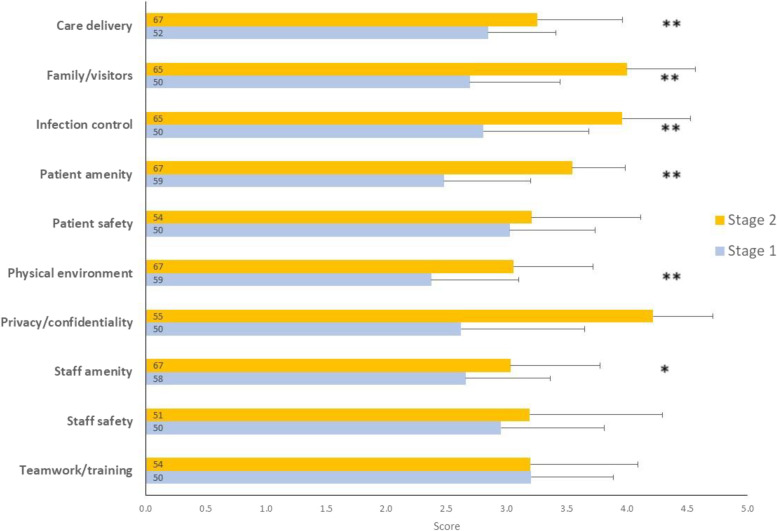
Means scores + 1 SD for ten sub-scales of the current ward layout, environment and facilities measured in four wards of the hospital during Stage 1 (darker) and Stage 2 (lighter). The sample size for each measure is included in the bars. ** *P* ≤ 0.001, * *P* ≤ 0.01)

Mean scores for the ten sub-scales of the ward layout, environment and facilities are presented for Stage 1 and Stage 2 in Fig. 1. Care delivery, family/visitors’ experience, infection control, patient amenity and physical environment and staff all increased significantly, indicating a more positive environment in Stage 2. There were no differences between Stage 1 and Stage 2 for patient safety, staff safety or teamwork/training.

### Comparison of nursing staff interviews between stage 1 and stage 2

Stage 2 explored nursing staffs’ experience of single-bed room environments and compared these with nursing staffs’ views before the move to all single-bed room hospital. Four themes are presented: Isolation from colleagues; Patient safety including visibility, falls risk and infection control; Wasting time and finding nursing staff and Patient privacy and confidentiality (See Table 2).

**Table 2:** Comparison of nursing staff interviews between Stage 1 and Stage 2

Theme	Stage 1 Quotes	Stage 2 Quotes
Isolation from colleagues	*The main thing should be about team work. One nurse should not be left to one room …. All the nurses should be working together. (Nurse Interview 10)*	*Not having a central tearoom in the wing I think impacts staff, it has had a negative effect. You have to walk to get there on your break, so for us the staff room is all the way down in F wing, … I think that has a negative effect from a morale point of view, they don’t get to sit in a tearoom, chat and debrief one another (Nurse Interview 6)* *I think the camaraderie and the way we all worked together as a team has changed, the change is partly because of the environment that we’re working in. We don’t work as a team as well as we did in the old hospital. (Nurse Interview 2)*
Patient safety including visibility, falls risk and infection control	*The six-bed bay is really cluttered, so a lot of trip hazards. Just physically trying to find the space to get in the right position to help a patient up that can be a challenge. (Nurse Interview 6)* *We have bays of four patients… you can see what’s going on. I think you provide a better service, because patients who are at high falls risk, you’re in there and you can see… and you can stop it before it happens. (Nurse Interview 11)* *I think being all single rooms, there’s going to be a lot more falls, because we can’t see as many people at once. So I think there’s going to be a lot more one-to-one nurse specials if people are falling. (Nurse Interview 14)* *The fact that each patient will have their own bathroom, so I think that will decrease our rate of infections. (Nurse Interview 5)*	*To see the patient is a bit difficult because of the layout… a lot of times patients will close their door, but for us closing doors, is not a good idea because we want to see them... (Nurse Interview 1)* *When you’re with one patient and the call bell goes or something happened to another patient, because you’re in the room with one patient it’s hard to hear or know what’s going on with other patients (Nurse Interview 3)* *Not every single room, even if it’s close to a desk, is high visibility because the doors are facing the wrong way so you can’t see into the room from the desk (Nurse Interview 11).* *It is lovely that patients’ have their own toilet but … I find that patients can fall quite easily in a bathroom and you don’t even know they’re in there (Nurse Interview 2)* *… I feel like overall in terms of infection control for the patient, it’s good (Nurse Interview 8)*
Wasting nursing time	*Finding equipment that works isn’t always easy sometimes. You can walk up and down the ward to find a blood pressure machine that’s actually been plugged in and is charged or a stethoscope or a manual blood pressure machine that the pump is working properly. (Nurse Interview 2)* *… Sometimes you can come on and between the early and the late you could move six patients. We don’t move them for nothing. It is genuine reasons. Sometimes it’s reasons because of the layout. They need a side room. (Nurse Interview 8)*	*..the way it’s set out you can’t find staff, you don’t know where anyone is, you can tend to do a lot more walking, I can walk around and around and around in circles looking for staff …. (Nurse Interview 2)*
Patient privacy and confidentiality	*Yeah, they will be more comfortable in a single room with privacy, confidentiality, maybe more room and less noise. (Nurse Interview 10)* *In a single room, handing over is going to be private because you’re not having people in a bay listening (Nurse Interview 16)* *Somebody who’s got limited mobility its difficult they have a bit of a walk. Single rooms they will be closer to the toilet it will be better more private (Nurse Interview 9)*	*It’s easier to have a conversation in a single room, facilitating more family meetings within that patient’s room rather than previously when we’d have to find a room to have sensitive conversations… (Nurse Interview 11)* *I think the good side is privacy. Sometimes when you’re having certain conversations with patients, discussing living situations to discharge them to or even doing some clinical procedures a curtain would not quite suffice (Nurse Interview 3)*

#### Isolation from colleagues

An issue raised by nursing staff in Stage 2, not mentioned in Stage 1 was a sense of being isolated from their colleagues with less interaction due to working in single rooms and not seeing each other in ward staff tearooms. In the previous hospital, wards had a tearoom where staff would gather during breaks.

#### Patient safety including visibility, falls risk and infection control

Previously, interviewed nurses liked open bays because multi-bed rooms allowed nursing staff to directly observe patients. After the move nurses reported concern about a lack of visibility of patients because of the layout of the wards did not provide a direct view through the door of the single-bed room.

In Stage 1, nurses were very concerned that the falls rate would increase in single-bed room environment due to a lack of patient visibility. This concern continued for some nursing staff in Stage 2. Lack of visibility made some nursing participants feel uneasy about their responsibility to prevent falls, because they often could not hear or see the patient. Therefore, techniques to monitor patients more frequently became a priority with increased walking between rooms to view patients, and allocation of rooms near the nurses’ station for high-risk patients. Reassuringly, nursing staff indicated they have not noticed a significant increase in falls.

Nurses previously indicated they expected infection control to be improved in single-bed room hospital when compared to mixed-bed rooms, and Stage 2 interviews suggests this perception remained. However, COVID-19 impacted on MRSA testing due to high demand for COVID swabs, so nurses’ perceptions may not be a true reflection of infection control rates. Furthermore, increased hygiene requirements implemented due to COVID-19 health orders and restrictions may have significantly impacted this observation.

#### Wasting nursing time

In Stage 1, nurses reported that looking for missing/misplaced case note or medical devices, and moving patients beds around the ward wasted their time. In the new hospital environment electronic case notes were used, which prevented wasting time as described in Stage 1. However, time wasting and increased walking during a shift were reported by nursing staff in the new hospital layout, when looking for co-workers, who were not easily found in 100% single room environment. Presence buttons/bells in each patient room (indicating a member of staff was in that single room), when used, were deemed helpful to locate colleagues. It was reported that quite often nurses simply forgot to turn presence button/bells on when entering patients’ rooms. Nursing staff in Stage 2 did not indicate any issues moving patient beds around to accommodate patients’ needs on single-bed wards compared to Stage 1. Therefore, the move to single-bed room environment appeared to have reduced time undertaking constant patient bed movements.

#### Patient privacy and confidentiality

A lack of privacy and confidentiality for patients were recognised as a disadvantage of open bays in Stage 1. Nursing staff saw the value of single-bed rooms in providing a much quieter private environment for patients (and visitors), and anticipated patients would appreciate improved privacy and confidentiality, contributing to maintaining dignity and comfort. However, nurses could also foresee that older patients in particular may feel more isolated and lonely, increasing levels of anxiety. Certainly, most of these themes were repeated in the Stage 2 analysis. Benefits of nursing staff being able to provide nursing care to patients in a discrete environment, enabling confidential conversations in privacy and without interruptions were acknowledged.

### Comparison of patient interviews between stage 1 and stage 2

The interview data explored patients’ experience of single-bed room environments, before and after the move to the all single room hospital. Three major themes are discussed: Being cared for; Patients privacy, noise and isolation and improved visitor experience (Table 3).

**Table 3:** Comparison of patient interviews between Stage 1 and Stage 2

Theme	Stage 1 Quotes	Stage 2 Quotes
Being cared for	*The people here look after you. People, it’s like - for me, it’s all about that. (Patient Interview 13)* *They can see in the rooms without actually having to come to the door. So I feel safe. (Patient Interview 14)*	*What matters the most? Well, I suppose the friendliness of the staff…. (Patient Interview 9)* *Best part of the experience? The staff have been very nice (Patient Interview 7)*
Patient privacy, noise and isolation	*You do get sick of sharing the same bathroom with somebody else. (Patient Interview 1)* *There is noise, but I don’t know whether you can stop that. People have got to push trolleys and beds and so it goes on. Noise is what I imagine the hospital to be. (Patient Interview 9)* *You do get down a little bit sometimes. I was like that myself this morning. You get a bit lonely and if you haven’t got interactions with other patients, you’ve got nothing, have you? (Patient Interview 7)* *Doctors always shut the curtains and they automatically think your sound proofed, no one can hear. You try not to listen so confidentiality would be another bonus of single rooms. (Patient Interview 5)* *I can see the activity here and I prefer that. (Patient Interview 12)*	*Obviously, the privacy that single room gives you. You go can use the toilet, the shower’s yours (Patient Interview 4)* *The nursing staff at night are so quiet. It’s just lovely. Once they close the doors it’s quiet. So I had no problems getting to sleep. It’s been very pleasant…. (Patient Interview 1)* *… in some ways it sort of let me be on my own, do my own thing. But also, it has isolated me. (Patient Interview 12)*
Improved visitor experience	*Well I think it would be quieter and perhaps you could have more opportunity to discuss personal things with visitors. Sometimes they have to discuss personal details so I think being in a single room would be good. (Patient Interview 6)*	*They have offered a bed for my husband, who’s coming over tomorrow. He can sleep here if he wishes (Patient Interview 5)*

#### Being cared for

Patient participants in both the Stage 1 and Stage 2 indicted that the two most important issues over any environmental factors were ‘being cared for’ and ‘the nursing staff relationship with patients’. Participants in Stage 2 appreciated the significant benefits of the new hospital environment, in particular the clean and modern look of the ward and outside views, which contributed to a positive experience.

#### Patient dignity, noise, and isolation

Stage 1 findings suggested privacy and dignity relied on the use of curtains, however, conversations could still be heard. Patient participants in Stage 1 recognised that curtains in a shared room/bay did not provide them with confidentiality, and as such participants in Stage 1 suggested the move to single-bed rooms would offer dignity and confidentiality. Single-bed room amenities met these anticipated patients’ expectations.

Stage 1 participants mentioned high noise levels consistently affected their ability to sleep, but accepted this to be a normal part of a patient’s hospital experience in shared bed environment. A move to single-bed rooms met expectations set out in Stage 1 providing a much quieter and restful environment for patients.

In Stage 1 some patients preferred sharing a room, allowing patients to access support networks and therapeutic social connection. However, this was not always a positive experience when sharing a room with intrusive noisy behaviours. Being in a single-bed room was anticipated to have benefits for those who liked to be on their own as well as the potential negative experience for some patients’ who may feel isolated. The potential to feel isolated in a single bed-room environment was mentioned by participants in Stage 2.

#### Improved visitor experience

Patient participants in Stage 1 anticipated the move to a single-bed room would be largely beneficial for patients who received a lot of visitors. In Stage 2, patient participants noted that not only was it a more private experience for visitors, participants also appreciated family members were able to stay overnight on daybeds, providing support and companionship (prior to COVID pandemic and subsequent visitor restrictions).

## Discussion

This paper presents a comparison of nursing staff and patients experience prior to a move to 100% single-bed room hospital in 2016 (Stage 1) and actual experiences after the move in 2021 (Stage 2). Survey and interview data offered insights into advantages and disadvantages of the all single-bed room design, adding to the discourse on nurses and patients experience.

Privacy was the main advantage of all single-bed rooms identified in-line with expectations during interviews, and this sub-scale not only scored highest in the Stage 2 survey but also demonstrated the largest increase from Stage 1 values. The value of single-bed rooms contributing to patients’ privacy, confidentiality, dignity and comfort was highlighted by both nurses and patients. For many patient participants in Stage 2, one of the most significant benefits of the move to single-bed rooms was access to their own bathroom and toilet offering improvement in convenience, and in maintaining privacy and dignity supporting previous work by Maben et al. [6]. From a patients’ perspective in Stage 1, sharing bathrooms was not generally liked, but was accepted as part of their hospital experience. The benefit of privacy and confidentiality for both patients and nurses were consistent with findings from other single-bed room studies [4, 6, 11–13].

Patients’ and nurses’ experiences of multi bed and single-bed room environments shared a number of interconnected themes. Patient participants in both the Stage 1 and 2 interviews suggested that the two most important issues over any environmental factors were ‘being cared for’ and ‘the nursing staff relationship with patients’. In comparison, nurses’ interviews revealed that the most important issues impacting their practice and patient care included ‘patient and nursing staff visibility and safety’. As predicted in Stage 1, our data in Stage 2 confirmed that nurses experienced a lack of visibility of patients, which influenced their workload and workflow. Donetto et al. [14] and Maben et al. [6] found that a lack of visibility does impact how nurses care for patients. Visibility of patients’ relates to the ongoing informal observation by nurses as they go about their work. In open bed bays, nurses continually assess patients’ behaviour and health status at a glance and respond quickly to any emergency. Other patients are part of that ongoing observation of each other, calling out to nurses if they become concerned. This continuous visibility is removed depending on the configuration of the single rooms, the location of doorways and inclusion of observation windows. Nurses in Stage 2 reported that the position of the doorways and doors prevented them looking directly into the room as they walked past. This reduced their visibility of patients’ as well as quickly finding their colleagues, which in turn increased walking distances, wasted time and influenced their workflow.

Our data did not support Søndergaard et al. [4] findings that patients reporting fears of being overlooked by nurses and not regularly monitored or that nursing care became more task driven, even though the environment increased privacy for deeper communication with patients. Supporting Maben et al. study [6] nursing staff concerns for patient safety and sense of responsibility for patients’ welfare behind closed doors remained strong in our Stage 2 data, with particular emphasis on falls risk.

The most divergent views in both this study and the literature [4] are in relation to patients’ personal preferences for single-bed room accommodation. The design of single-bed room accommodation benefitted some patient preferences over others. In Stage 1 some patients reported that they liked the company of others in the bay environment while others did not. In both Stages of this study, patients and nursing staff expressed some concerns that it was possible for single-bed room accommodation to leave patients feeling isolated and lonely. Specifically, nurses interviewed in Stage 2 noted that some types of patients (particularly the elderly and/or confused) were in need of companionship and support (also, they preferred to see what was going on). Interestingly, Maben et al. [6] found that almost half of the men interviewed in their study preferred multi-bedded accommodation, rather than single-bed rooms, showing a gender differences in preferences. To address the divergent views and needs of both patients and nursing staff, nursing staff suggest incorporating both multi-bed and single-bed rooms in new hospital designs to accommodate different patient preferences and enhance visibility of vulnerable people. Previous academic work in support of a mixed-room layout for age, cultural and medical reasons corroborates this [6, 15–20].

For some patient participants, single-bed rooms met the expectations set out in Stage 1 of this study [1], in that they provided a much quieter and restful environment and demonstrated improvement in the ‘Patient amenity’ sub-scale in Stage 2. Previously, multi-bed wards and a cramped environment contributed to a high level of general background noise and disruptive sources of noise especially at night when patients were trying to sleep. In Stage 2, nurses acknowledged that the single-bed rooms are conducive to better sleep due to fewer sounds within the single bed-room. This view was supported by findings that single-bed rooms provide a more restful environment due to less noise and did improve patients’ sleep [6, 11, 18, 21, 22].

In Stage 1, nursing staff indicated an anticipated concern that a move to an all single-bed room might negatively impact on the team and how they worked together, however, the ‘Teamwork/Training’ sub-scale remained the same in Stage 1 and Stage 2. Yet the Stage 2 nursing staff interview data indicated some aspects where participants felt isolated from their colleagues, because they were often working on their own and could not readily locate their colleagues when required. These feelings of being isolated in their practice were reported by Maben et al. [6] and supported by Søndergaard et al. [4], where the notion of ‘lonely practice’ was identified. The sense of isolation discussed by Maben et al., [6] was linked to nurses feeling deprived of feedback and opportunities to learn from their colleagues, and identified this as a disadvantage of single bed-room accommodation. Donetto et al. [14] also discussed the loss of teamwork associated in a single-bed room environment. The notion of ‘lonely practice’ for nurses, working in the all single-bed room environment does warrant further research into models of nursing care, because it has implications for nursing staff safety, (perceptions of which however did not change from Stage 1 to Stage 2), mentoring, sense of a supportive collegial environment and retention, as well as increased potential for undetected poor practice.

### Limitations

Survey response rate was 14% for final analysis including demographics, but a little higher (23%) for some sub-scales. Lack of diversity in patients interviewed in terms of age groups, patient vulnerability (dementia or very unwell patients) and ethnic backgrounds, all of whom, may have had a different experience, is also a limitation. The newness of the hospital environment may also have influenced the experience of the single-bed room by patients, where no aging of the physical surroundings were evident. This study also lacks the data on patient falls and infection rates pre and post move to the new hospital so it is not possible to ascertain if nurses’ perceptions of no increase in falls and reduced infection rates is evidenced. Data collection timeframe of approximately 4 years should also be acknowledged as a limitation. The COVID-19 pandemic compromised the data collection process due to external visitor restrictions that impacted access to patients and nursing staff for interviews in the hospital.

## Conclusion

Patient and nursing staff experiences are often interdependent, and the implications of single-bed room accommodation is complicated. Patient participants reported a high level of satisfaction with their experience staying in single-bed rooms, which may partly be due to the newness of the hospital surroundings. Nursing staff acknowledged advantages and disadvantages of single-bed layout and noted the design benefited some people (i.e. those who are younger and/or independent) and not others (i.e. older and confused patients). Future impacts on the health system will continue to affect hospital design, which must consider nurses working needs as well as patient safety and comfort.

## Supplementary Information

**Additional file 1:** **Supplementary Material S1.** Nurse Survey.

**Additional file 2:** **Supplementary Material S2.** Nurse Interview schedule.

**Additional file 3:** **Supplementary Material** **S3.** Patient Interview Schedule.

## Data Availability

The datasets generated and analysed during the current study are not publicly available due to confidential nature of the participant data but are available from the corresponding author on reasonable request.
