# Supplementary material for: Comparison of experiences of nursing staff and patients before and after move to 100% single-bed room hospital in Australia: mixed methods
Source: BMC Health Serv Res. 2023 Jan 25;23:81. doi: 10.1186/s12913-023-09073-8 (PMC9875193; doi:10.1186/s12913-023-09073-8)
Supplement: Supplementary file 1 — Additional file 1: Supplementary Material S1. Nurse Survey. [file 12913_2023_9073_MOESM1_ESM.pdf]

SECTION 1: WARD LAYOUT, ENVIRONMENT AND FACILITIES

I understand the purpose of the survey, that completion of the survey is voluntary and my identity will not be recorded. I agree to take part. Yes □ [please tick yes to continue] No □

A. To what extent do you agree or disagree with the following statements about the ward layout, environment, facilities and information and communications technology (ICT) on this ward?

Please circle the number from 1 (Strongly disagree) to 5 (Strongly agree) that best matches your personal view.

	**Statement**	**Strongly disagree**	**Dis-agree**	**Neither agree nor disagree**	**Agree**	**Strongly agree**
1	The supplies, consumables and equipment needed to care for patients are always available on the ward	1	2	3	4	5
2	Staff toilet facilities are adequate	1	2	3	4	5
3	Patient toilets and bathrooms are a good size and allow for easy access	1	2	3	4	5
4	It is easy for staff who are new to the ward to find their way about	1	2	3	4	5
5	Staff have regular access to a designated rest area (e.g. tea room)	1	2	3	4	5
6	Most patients are able to see staff from their bed	1	2	3	4	5
7	It is easy to keep patient care areas clean	1	2	3	4	5
8	Space at patients' bedsides is sufficient for staff to provide care with ease	1	2	3	4	5
9	Lighting levels in patient care areas are easy to adjust	1	2	3	4	5
10	Staff changing and locker facilities are adequate	1	2	3	4	5
11	It is easy to keep patient care areas quiet (e.g. around the patient room or bay)	1	2	3	4	5
12	The ward layout makes it easy to monitor (keep an eye on) patients	1	2	3	4	5
13	The location of staff workstations enables staff to remain close to patients	1	2	3	4	5
14	Most patients have a window view of a natural setting / scene from their bed	1	2	3	4	5
15	It is easy for patients, families and visitors to find their way about within the ward	1	2	3	4	5
16	It is easy for patients to get to the toilet / bathroom (alone or assisted)	1	2	3	4	5
17	Ventilation (air flow) in patient care areas is adequate	1	2	3	4	5
18	The availability of computers and IT equipment is adequate	1	2	3	4	5
19	The location and layout of the dirty utility room helps reduce the risk of spillages and cross contamination	1	2	3	4	5
20	It is easy to chart / document care close to the patient	1	2	3	4	5
21	There is adequate space at the nurses station(s)	1	2	3	4	5
22	Storage space for sterile supplies, consumables and equipment is adequate	1	2	3	4	5
23	The ward design / layout is helpful for isolating patients with infections	1	2	3	4	5
24	There is adequate natural light (daylight) for patients	1	2	3	4	5
25	The staff rest area aids relaxation and recuperation (e.g. tea room)	1	2	3	4	5
26	There are sufficient toilets and bathrooms for patients on this ward	1	2	3	4	5
27	The ward layout helps to minimise walking distances for staff	1	2	3	4	5
28	The ward design / layout minimises the need to move patients within the ward	1	2	3	4	5

	Statement	Strongly disagree	Dis-agree	Neither agree nor disagree	Agree	Strongly agree
29	It is easy to adjust the temperature in patient care areas	1	2	3	4	5
30	There is adequate space for patients to move about the ward if appropriate	1	2	3	4	5
31	The medication storage and preparation area is adequate	1	2	3	4	5
32	The number and location of clinical hand wash basins supports good hand hygiene	1	2	3	4	5
33	The supplies, consumables and equipment needed to care for patients are easily accessible	1	2	3	4	5
34	There is adequate space for family members and visitors at the bedside	1	2	3	4	5
35	There is adequate storage for patients' clothes and belongings	1	2	3	4	5
36	There is adequate natural light (daylight) for staff	1	2	3	4	5
37	Facilities provided for patients and visitors (e.g. day room) are adequate	1	2	3	4	5

B. Thinking about the current ward layout, environment, facilities and information and communications technology (ICT) on this ward, in your opinion how helpful or unhelpful are they for the following?

Please circle the number from 1 (Very unhelpful) to 5 (Very helpful) that best matches your personal view.

	Statement	Very unhelpful	Un-helpful	Neither helpful nor unhelpful	Helpful	Very helpful
1	Obtaining assistance (hands-on help) from colleagues with patients, procedures, equipment, IT or locating people or items	1	2	3	4	5
2	Safety and security of patients	1	2	3	4	5
3	Communication between nursing staff and patients	1	2	3	4	5
4	Monitoring new/junior team members/staff	1	2	3	4	5
5	Communication between nursing staff and doctors	1	2	3	4	5
6	Ability of staff to spontaneously discuss issues of care	1	2	3	4	5
7	Ability of staff to keep each other updated about general running issues on ward (e.g. staffing, patient dependency, number and status of patients)	1	2	3	4	5
8	Privacy for patients during personal care/washing / toileting	1	2	3	4	5
9	Discussing patient care with colleagues	1	2	3	4	5
10	Knowing when other staff might need a helping hand	1	2	3	4	5
11	Preventing and controlling hospital-acquired infections	1	2	3	4	5
12	Informal learning / learning from colleagues	1	2	3	4	5
13	Minimising the risk of medication errors	1	2	3	4	5
14	Being aware of general running issues on ward (e.g. staffing, patient dependency, number and status of patients)	1	2	3	4	5
15	Maintaining patient confidentiality	1	2	3	4	5
16	Social interaction among ward staff	1	2	3	4	5
17	Minimising the risk to staff of moving and handling injuries	1	2	3	4	5
18	Participation of family members in patient care	1	2	3	4	5
19	Overall comfort of patients	1	2	3	4	5
20	Minimising the risk to patients of physical and/or verbal abuse from other patients / visitors	1	2	3	4	5
21	Responding to patient calls for assistance	1	2	3	4	5
22	Minimising the risk to staff of slips, trips and falls	1	2	3	4	5
23	Privacy for patients during examination	1	2	3	4	5

	Statement	Very unhelpful	Un-helpful	Neither helpful nor unhelpful	Helpful	Very helpful
24	Social contact between patients	1	2	3	4	5
25	Minimising the risk to staff of physical and/or verbal abuse from patients / visitors	1	2	3	4	5
26	Ability of staff to deliver high quality care for all patients	1	2	3	4	5
27	Staff spending time with patients	1	2	3	4	5
28	Safety and security of staff	1	2	3	4	5
29	Minimising the risk of falls and injury to patients	1	2	3	4	5
30	Privacy for patients when giving medical history or being advised	1	2	3	4	5
31	Patient sleep and rest	1	2	3	4	5
32	Finding a staff member	1	2	3	4	5
33	Patient interaction with visitors	1	2	3	4	5
34	Minimising the risk to staff of needlestick and sharps injuries	1	2	3	4	5
35	Obtaining advice from colleagues relating to a skill or clinical knowledge	1	2	3	4	5

C. What two things do you think would most improve the current ward environment for staff? Please list in order of priority with most important first.

1.

2.

D. What two things do you think would most improve the current ward environment for patients? Please list in order of priority with most important first.

1.

2.

SECTION 2: EXPERIENCE OF 100% SINGLE ROOMS

A. In comparison to a ward with multi-bed bays, how much better or worse is it working in a ward with all single-bed rooms in regard to the following?

Please circle the number from 0 (Don't know / unsure) to 5 (Much better) that best matches your personal view.

	Statement	Don't know/ unsure	Much worse	Worse	No different	Better	Much better
1	Minimising the risk of falls and injury to patients	0	1	2	3	4	5
2	Minimising the need to move patients within the ward	0	1	2	3	4	5
3	Keeping patient care areas clean	0	1	2	3	4	5
4	Communication between nursing staff and patients	0	1	2	3	4	5
5	Participation of family members in patient care	0	1	2	3	4	5
6	Responding to patient calls for assistance	0	1	2	3	4	5
7	Preventing and controlling hospital-acquired infections	0	1	2	3	4	5
8	Patient sleep and rest	0	1	2	3	4	5

	Statement	Don't know/ unsure	Much worse	Worse	No different	Better	Much better
9	Ease of taking patients to the toilet / bathroom	0	1	2	3	4	5
10	Ability of staff to deliver high quality care for all patients	0	1	2	3	4	5
11	Maintaining patient confidentiality	0	1	2	3	4	5
12	Minimising the risk to staff of physical and/or verbal abuse from patients / visitors	0	1	2	3	4	5
13	Knowing when other staff might need a helping hand	0	1	2	3	4	5
14	Ability of patients to see staff	0	1	2	3	4	5
15	Patient privacy	0	1	2	3	4	5
16	Patient interaction with visitors	0	1	2	3	4	5
17	Minimising staff walking distances on the ward	0	1	2	3	4	5
18	Minimising risk to patients of physical and/or verbal abuse from other patients/ visitors	0	1	2	3	4	5
19	Social contact between patients	0	1	2	3	4	5
20	Overall comfort of patients	0	1	2	3	4	5
21	Minimising the risk of medication errors	0	1	2	3	4	5
22	Staff spending time with patients	0	1	2	3	4	5
23	Monitoring (keeping an eye on) patients	0	1	2	3	4	5

SECTION 3: YOUR MOST RECENT SHIFT ON THIS WARD

A. Which best describes the most recent shift you worked on this ward?

1☐ Early 2☐ Late

3☐ Long day 4☐ Night

5☐ Other: please specify:

B. On which day of the week did your most recent shift on this ward begin?

1☐ Mon - Fri 2☐ Sat 3☐ Sun

C. How many hours did you work on your most recent shift on this ward (excluding meal breaks)?

☐ hours

D. On your most recent shift on this ward did you work beyond your contracted hours?

1☐ Yes 2☐ No

E. In total, how many patients were you directly responsible for on the most recent shift you worked on this ward?

☐ patients

F. Is this number of patients typical of your usual workload for this shift/day on this ward?

1☐ Less 2☐ Typical 3☐ More

G. How many <u>other</u> staff (not including yourself) were also looking after these patients?

☐ staff

H. Of the patients you were directly responsible for on your most recent shift, how many required the following?

		Number
1	Assistance with all activities of daily living	
2	Hourly or more frequent monitoring or treatments	

I. How would you describe your role in caring for patients on your most recent shift on this ward?

Please mark the one option that fits best.

1☐ I provided most care myself

2☐ I supervised the care given by others and provided some myself

3☐ Most direct care was provided by others

Note: This survey was adapted with approval from Maben J, Griffiths P, Penfold C, Simon M, Pizzo E, Anderson J, Robert G, Hughes J, Murrells T, Brearley S, & Barlow J. Evaluating a major innovation in hospital design: workforce implications and impact on patient and staff experiences of all single room hospital accommodation. Health Services and Delivery Research. 2015 pg 215: doi:10.3310/hsdr03030

J. Overall, how satisfied are you with the quality of care that you provided to patients on your most recent shift on this ward?

0 Not applicable (did not provide direct care) | 1 Very dissatisfied | 2 Dissatisfied | 3 Neither satisfied nor dissatisfied | 4 Satisfied | 5 Very satisfied

K. On your most recent shift on this ward, how often did you perform the following tasks?

Please circle the number from 0 (Never) to 2 (Three or more times) that best matches your activity.

		Never	Once or twice	Three or more times
1	Delivering and retrieving food trays	0	1	2
2	Arranging discharge referrals and transportation	0	1	2
3	Taking routine blood samples from patients	0	1	2
4	Escorting patients within hospital	0	1	2
5	Cleaning patient rooms and equipment	0	1	2
6	Obtaining supplies or equipment	0	1	2
7	Answering phones, clerical duties (not related to patient care)	0	1	2

L. How satisfied were you with how you were able to perform the following on your most recent shift on this ward?

Please circle the number from 1 (Very dissatisfied) to 5 (Very satisfied) that best matches your personal view. Please circle 0 (Not applicable) if a task was not part of your role on your most recent shift.

		**Not applicable**	**Very dis-satisfied**	**Dis-satisfied**	**Neither satisfied nor dissatisfied**	**Satisfied**	**Very satisfied**
1	Physical care of patients (e.g. treatments and procedures)	0	1	2	3	4	5
2	Emotional care of patients	0	1	2	3	4	5
3	Monitoring / recording patients' observations	0	1	2	3	4	5
4	Pain management	0	1	2	3	4	5
5	Assisting patients with activities of daily living	0	1	2	3	4	5
6	Educating / teaching patients and family	0	1	2	3	4	5
7	Medication administration	0	1	2	3	4	5
8	Preparing patients for admission / discharge	0	1	2	3	4	5
9	Care planning / coordination	0	1	2	3	4	5
10	Documenting care	0	1	2	3	4	5

SECTION 4: JOB SATISFACTION, TEAMWORK AND SAFETY

A. Overall, how satisfied are you with your job?

1 Very dissatisfied | 2 Dissatisfied | 3 Neither satisfied nor dissatisfied | 4 Satisfied | 5 Very satisfied

B. To what extent do you agree or disagree with the following statements about teamwork on this ward?

Please circle the number from 1 (Strongly disagree) to 5 (Strongly agree) that best matches your personal view.

		**Strongly disagree**	**Dis-agree**	**Neither agree nor disagree**	**Agree**	**Strongly agree**

1	Staff input is well received on this ward	1	2	3	4	5
2	Decision making on this ward uses input from relevant staff	1	2	3	4	5
3	The doctors and nurses on this ward work together as a well coordinated team	1	2	3	4	5
4	Disagreements on this ward are resolved appropriately (i.e. not who is right, but what is best for the patient)	1	2	3	4	5
5	It is easy for staff on this ward to ask questions when there is something that they do not understand	1	2	3	4	5
6	I have the support I need from other staff to care for patients	1	2	3	4	5
7	I know the first and last names of all the staff I worked with during my last shift	1	2	3	4	5
8	Important issues are well communicated at shift changes	1	2	3	4	5
9	Briefings are common on this ward	1	2	3	4	5

C. How satisfied are you with quality of communication that you experience with each of the following staff groups on this ward?

Please circle the number from 1 (Very dissatisfied) to 5 (Very satisfied) that best matches your personal view. Please circle 0 (Not applicable) if your role does not require you to communicate with a particular staff group.

		Not applicable	Very dis-satisfied	Dis-satisfied	Neither satisfied nor dissatisfied	Satisfied	Very satisfied
1	Doctors	0	1	2	3	4	5
2	Registered nursing staff	0	1	2	3	4	5
3	Enrolled nursing	0	1	2	3	4	5
4	Assistant in nursing	0	1	2	3	4	5
5	Allied health professionals (e.g. PT, OT, SpT, Dietician)	0	1	2	3	4	5

D. To what extent do you agree or disagree with the following statements about safety on this ward?

Please circle the number from 1 (Strongly disagree) to 5 (Strongly agree) that best matches your personal view.

	Statement	Strongly disagree	Dis-agree	Neither agree nor disagree	Agree	Strongly agree
1	I am encouraged by my colleagues to report any patient safety concerns I may have	1	2	3	4	5
2	The culture on this ward makes it easy to learn from the errors of others	1	2	3	4	5
3	I receive appropriate feedback about my performance	1	2	3	4	5
4	Medical errors are handled appropriately on this ward	1	2	3	4	5
5	I know the proper channels to which I should direct questions regarding patient safety	1	2	3	4	5
6	The levels of staffing on this ward are sufficient to handle the number of patients	1	2	3	4	5
7	I would feel safe being treated as a patient on this ward	1	2	3	4	5
8	Hospital management does not knowingly compromise the safety of patients	1	2	3	4	5
9	This organisation is doing more for patient safety now than it did one year ago	1	2	3	4	5
10	Leadership is driving us to be a safety-centred organisation	1	2	3	4	5
11	My suggestions about safety would be acted upon if I expressed them to management	1	2	3	4	5

E. During the last 12 months have you been injured or felt unwell as a result of the following on this ward?

1 Moving and handling 1□ Yes 2□ No

2 Needlestick and sharps injuries 1 Yes 2 No

3 Slips, trips or falls 1 Yes 2 No

F. In the last 12 months have you personally experienced physical violence, harassment, bullying or abuse on this ward?

1 From patients or their partners /relatives /visitors 1 Yes 2 No

2 From colleagues 1 Yes 2 No

G. To what extent do you agree or disagree with the following statements about working on this ward?

Please circle the number from 1 (Strongly disagree) to 5 (Strongly agree) that best matches your personal view

	**Statement**	**Strongly disagree**	**Dis-agree**	**Neither agree nor disagree**	**Agree**	**Strongly agree**
1	I often feel under a lot of pressure at work	1	2	3	4	5
2	I worry a lot about my work outside working hours	1	2	3	4	5
3	My job is very stressful	1	2	3	4	5

SECTION 5: BACKGROUND DETAILS

A. What is your gender?

1 Female 2 Male

B. What is your age?

1 16-20 2 21-30 3 31-40
4 41-50 5 51-65 6 66+

C. How many hours a week are you contracted to work?

1 Up to 29 hours 2 30 or more hours a week

D. Do you regularly work outside your contracted hours?

1 Yes 2 No

E. Which shifts have you worked in the last month?

Please tick all that apply.

1 Early 2 Late

3 Long day 4 Night

5 Other: please specify:

F. What is your occupational group?

1	Registered Nurse (RN)	2	Enrolled Nurse (EN)
3	Assistant In Nursing (AIN)	4	Other: please specify

G. Approximately, how many years have you worked?
Please enter number of years for each of the following:

		Years			Years
1	In your current occupational group (i.e. as RN, EN, AIN)		2	In this specialty	
3	In the RAH		4	On this ward	

H. What is your current pay band?

1 Level 1 2 Level 2 3 Level 3

4 Level 4 5 Level 5

I. Did you undertake your initial nursing education in Australia?

1 Yes 2 No 3 Not applicable

J. What is your highest level of qualification?

1	No academic qualification	2	Year 12 (up to and including)
3	Certificate IV	4	Diploma
5	Bachelor of Nursing	6	Postgraduate qualification (Grad Dip, Masters, PhD)

K. Please use the space below and overleaf to write any additional comments you have about the ward layout, environment, facilities and information and communications technology (ICT) in the old and/or new hospital:

THANK YOU VERY MUCH FOR YOUR TIME AND EFFORT IN COMPLETING THIS QUESTIONNAIRE

Note: This survey was adapted with approval from Maben J, Griffiths P, Penfold C, Simon M, Pizzo E, Anderson J, Robert G, Hughes J, Murrells T, Brearley S, & Barlow J. Evaluating a major innovation in hospital design: workforce implications and impact on patient and staff experiences of all single room hospital accommodation. Health Services and Delivery Research. 2015 pg 215: doi:10.3310/hsdr03030
