# Supplementary material for: Comparison of experiences of nursing staff and patients before and after move to 100% single-bed room hospital in Australia: mixed methods
Source: BMC Health Serv Res. 2023 Jan 25;23:81. doi: 10.1186/s12913-023-09073-8 (PMC9875193; doi:10.1186/s12913-023-09073-8)
Supplement: Supplementary file 2 — Additional file 2: Supplementary Material S2. Nurse Interview schedule. [file 12913_2023_9073_MOESM2_ESM.pdf]

Topic Guide for Staff Interviews

Research objectives for staff interviews:
• Has the move to all single room accommodation affect staff experience and well being?
• How does the physical environment impact on the experience of staff providing care to patients and how does this influence staff ability and capacity to deliver high quality care
• Does it affect staff working practices and are these changed?
• Does it affect ability of staff to deliver high quality patient care?
• Are there advantages for staff of a move to all single room accommodation?
• Are there disadvantages for staff of a move to all single room accommodation?

Note on use of this topic guide:
We wish to encourage participants to discuss their views and experiences in an open way without excluding issues that may be of importance to individual participants and the study as a whole. Therefore, unlike a survey questionnaire or semi-structured interview, the questioning will be responsive to respondents' own experiences, attitudes and circumstances.
The following guide does not contain pre-set questions but rather lists the key themes and sub-themes to be explored with each participant. This allows the interviewer to formulate questions which are responsive to each individual participant. The topic guide does not include follow-up questions like `why', `when', `how', etc. as it is assumed that participants' contributions will be fully explored throughout in order to understand how and why views, behaviours and experiences have arisen. While all topics will be covered with each participant, the order in which issues are addressed and the amount of time spent on different themes will vary between participants.

1. Introduction
- Introduce self, School of Nursing
- Introduce research (funding, research design, outputs)
- Explain: confidentiality, tape recording, length of interview, nature of discussion (specific topics to address, but conversational in style, in your own words, no right or wrong answers), reporting and data storage/archiving
- Any questions
- Obtain written consent

2. Background
Aim: to gather background contextual information which may have a bearing on experiences and can be followed up and explored during interview
- Personal circumstances (grade of staff; where work; how long worked)
- Previous experience as member of staff elsewhere (and check whether worked in the previous hospital before move to new hospital)

3. Recent experience as member of staff in clinical service
Aim: to capture spontaneous reflections on recent experience and what aspects were important to participant
- What is it like working in ward area?
- Overall impression of ward and hospital
- Experience (best and worst aspects of experience)

Note: Adapted with permission from - Maben J, Griffiths P, Penfold C, Simon M, Pizzo E, Anderson J, Robert G, Hughes J, Murrells T, Brearley S, & Barlow J. Evaluating a major innovation in hospital design: workforce implications and impact on patient and staff experiences of all single room hospital accommodation. Health Services and Delivery Research. 2015 pg 207: doi:10.3310/hsdr03030

4. Staff experience relating to physical environment
Aim: to encourage participant to reflect spontaneously on the physical environment in relation to staff experience

- Positive aspects of physical environment (layout, environment, facilities)
- Negative aspects of physical environment (layout, environment, facilities)
- Affects different staff groups differently?

5. Ward layout, environment and facilities and direct care
Explore perceptions of impact of ward layout, environment, facilities and technology on direct care delivery and processes

- Space at bedside
- Ease of taking patients to toilets/bathrooms
- General communication/interaction with patient
- Education/teaching
- Patient privacy/confidentiality
- Mobilising patients
- Visibility of patients
- Awareness of general running issues on ward (e.g. staffing, patient dependency, number and status of patients)
- Nurse call system (responding to patient calls)
- Issues affecting time allocated to direct care (e.g. walking distances, location, availability and accessibility of supplies, consumables and equipment)

6. Ward layout, environment and facilities and communication/teamwork
Explore perceptions of impact of environment on different aspects of communication and teamwork

- Seeking advice / assistance
- Informal teaching / learning
- Monitoring new/junior colleagues
- Discussing patient care (formal and spontaneous)
- Visibility of staff/finding a staff member
- Location and design of nursing team stations
- Patient status board
- Communication systems/devices
- Confidentiality
- Keeping staff updated
- Staff coordination (e.g. team briefings/discussion)
- Communication between nursing staff and doctors
- Differences depending on time of day/night
- Social aspects of work and staff well being – social interaction/support/isolation, staff space and facilities

7. Ward layout, environment and facilities and documentation and medication tasks
Explore perceptions of impact of environment on documentation and medication tasks

- How ward layout affects work processes in relation to these
- Work stations (location, design, location, space etc.)
- IT availability and mobile solutions

Note: Adapted with permission from - Maben J, Griffiths P, Penfold C, Simon M, Pizzo E, Anderson J, Robert G, Hughes J, Murrells T, Brearley S, & Barlow J. Evaluating a major innovation in hospital design: workforce implications and impact on patient and staff experiences of all single room hospital accommodation. Health Services and Delivery Research. 2015 pg 207: doi:10.3310/hsdr03030

- Medication storage, preparation and administration

8. Ward layout, environment and facilities and staff and patient safety
Explore perceptions of impact of environment on staff and patient safety

- Moving & handling
- Slips, trips and falls (staff)
- Needlestick/sharps injuries
- Infection control (including hand hygiene, cleanliness of patient care areas)
- Security of staff and patients(risk of physical/verbal abuse from patient/visitors)

9. Patient experience relating to physical environment
Aim: to understand staff perceptions' of the patient experience relating to aspects of the physical environment

- Visibility of staff
- Privacy
- Security
- Communication and support from other patients
- Interaction with visitors
- Involvement of family in patient care
- Bed and other furniture (e.g. chair, bedside cabinet)
- Facilities (bathroom, day room, accessibility of these)
- Risk of falls and injury
- Entertainment/communication (television, radio, telephone)
- During the day (noise, lighting, temperature, view outside)
- At night (noise, lighting, temperature, quality of sleep)
- Any times / experiences when patients feel particularly uncomfortable / unsafe
- Affects different patients differently (e.g. young adults, confused/dementia patients)?

10. Suggestions for improvements
Aim: to obtain staff suggestions for what would improve the physical environment and close interview on a positive note

- Explore improvements for staff, patients and visitors
    - Ward layout
    - Ward environment (e.g. heating, lighting, ventilation)
    - Ward facilities
    - Technology (ICT)
    - Any other aspects of physical environment

- Anything else would like to add
