# Supplementary material for: Comparison of experiences of nursing staff and patients before and after move to 100% single-bed room hospital in Australia: mixed methods
Source: BMC Health Serv Res. 2023 Jan 25;23:81. doi: 10.1186/s12913-023-09073-8 (PMC9875193; doi:10.1186/s12913-023-09073-8)
Supplement: Supplementary file 3 — Additional file 3: Supplementary Material S3. Patient Interview Schedule. [file 12913_2023_9073_MOESM3_ESM.pdf]

Topic Guide for Patient Interviews

Research objectives for patient interviews:
• Has the move to all single room accommodation affected patient and carer experience and well being?
• Does it affect diverse patient groups (e.g. pregnant women, the elderly) differently?
• Are there advantages for patients of a move to all single room accommodation?
• Are there disadvantages for patients of a move to all single room accommodation?

Note on use of this topic guide:
We wish to encourage participants to discuss their views and experiences in an open way without excluding issues that may be of importance to individual participants and the study as a whole. Therefore, unlike a survey questionnaire or semi-structured interview, the questioning will be responsive to respondents' own experiences, attitudes and circumstances.
The following guide does not contain pre-set questions but rather lists the key themes and sub-themes to be explored with each participant. This allows the interviewer to formulate questions which are responsive to each individual participant. The topic guide does not include follow-up questions like `why', `when', `how', etc. as it is assumed that participants' contributions will be fully explored throughout in order to understand how and why views, behaviours and experiences have arisen. While all topics will be covered with each participant, the order in which issues are addressed and the amount of time spent on different themes will vary between participants.

1. Introduction

- Introduce self, School of Nursing
- Introduce research (funding, research design, outputs)
- Explain: confidentiality, tape recording, length of interview, nature of discussion (specific topics to address, but conversational in style, in your own words, no right or wrong answers), reporting and data storage/archiving
- Any questions
- Obtain (written) consent

2. Background

Aim: to gather background contextual information which may have a bearing on experiences and can be followed up and explored during interview

- Personal circumstances (main daytime activity, who live with)
- Previous experience as hospital in-patient

3. Recent experience as patient in clinical service

Aim: to capture spontaneous reflections on recent experience and what aspects were important to participant

- Length of stay
- Overall impression of ward and hospital
- Experience (**best** and **worst** aspects of experience)
- Satisfaction (how satisfied overall with care and treatment received)

Note: Adapted with approval from- Maben J, Griffiths P, Penfold C, Simon M, Pizzo E, Anderson J, Robert G, Hughes J, Murrells T, Brearley S, & Barlow J. Evaluating a major innovation in hospital design: workforce implications and impact on patient and staff experiences of all single room hospital accommodation. Health Services and Delivery Research. 2015: doi:10.3310/hsdr03030

4. Experience of being admitted to ward

Aim: to understand 'touch point' and significance for overall experience

- First impressions of ward environment (welcoming / unwelcoming, homely / clinical, friendly / frightening)
- Staff
- Ward (and layout of ward)
- Other patients
- Orientation to ward and facilities

5. Feeling comfortable

Aim: to understand aspects of the physical environment which influenced perceptions of comfort

- Impressions of ward environment over time
- General impact of environment on experience of feeling comfortable
- Bed and other furniture (e.g. chair, bedside cabinet)
- Facilities (toilets, showers/baths, day room)
- Ward layout (accessibility, noise, privacy etc.)
- Décor
- Entertainment/communication (television, radio, telephone)
- During the day (noise, lighting, ventilation, temperature)
- Views from bed, views from windows
- At night (noise, temperature, lighting, quality of sleep)
- Other patients
- Any times / experiences when felt particularly uncomfortable
- Any times / experiences when felt particularly comfortable

6. Feeling safe

Aim: to understand aspects of the physical environment which influenced perceptions of safety

- General impact of environment on experience of feeling safe
- Security of belongings
- Nurse call system / calling for assistance (ease or difficultly during day and night)
- Moving around the ward (any concerns re falls and injury)
- Visibility of staff
- Privacy, dignity, confidentiality
- Cleanliness and hygiene on the ward (including experience of staff hand washing)
- Any times / experiences when felt particularly unsafe
- Any times / experiences when felt particularly safe

7. Interaction with staff

Aim: to understand how role of physical environment in patients' interactions with staff

- General atmosphere on ward (friendly, unfriendly)
- Staff behaviour and how interacted with patients (who, for what reason, how often)
- Relationships with staff (explore for different staff groups: domestics; assistant/support workers; midwives/nurses; doctors)
- Positive experiences
- Negative experiences

Note: Adapted with approval from- Maben J, Griffiths P, Penfold C, Simon M, Pizzo E, Anderson J, Robert G, Hughes J, Murrells T, Brearley S, & Barlow J. Evaluating a major innovation in hospital design: workforce implications and impact on patient and staff experiences of all single room hospital accommodation. Health Services and Delivery Research. 2015: doi:10.3310/hsdr03030

- Ways in which physical environment made interactions easier / more difficult (privacy, dignity, confidentiality)

8. Interaction with visitors

Aim: to explore how the physical environment made visiting enjoyable / less enjoyable

- Visiting times and visitors
- Facilities for visitors (chairs, refreshments etc.)
- Staff interaction with visitors

9. Suggestions for improvements

Aim: to obtain patients' suggestions for what would improve the physical environment and close interview on a positive note

- Ward layout
- Other aspects of physical environment (noise, lighting, heating, ventilation)
- Facilities (toilets and bathrooms, day rooms, entertainment and telephones etc.)
- Visibility of staff
- Visitors
- Looking back now is there anything in particular that would have made your experience better/easier?
- How do you think we can improve patient experiences within these services?
- What matters most to you as a patient?
- Anything else would like to add

End interview

Note: Adapted with approval from- Maben J, Griffiths P, Penfold C, Simon M, Pizzo E, Anderson J, Robert G, Hughes J, Murrells T, Brearley S, & Barlow J. Evaluating a major innovation in hospital design: workforce implications and impact on patient and staff experiences of all single room hospital accommodation. Health Services and Delivery Research. 2015: doi:10.3310/hsdr03030
